# A novel diffusion‐tensor MRI approach for skeletal muscle fascicle length measurements

**DOI:** 10.14814/phy2.13012

**Published:** 2016-12-21

**Authors:** Jos Oudeman, Valentina Mazzoli, Marco A. Marra, Klaas Nicolay, Mario Maas, Nico Verdonschot, Andre M. Sprengers, Aart J. Nederveen, Gustav J. Strijkers, Martijn Froeling

**Affiliations:** ^1^ Department of RadiologyAcademic Medical CenterAmsterdamthe Netherlands; ^2^ Orthopedic Research LabRadboud UMCNijmegenthe Netherlands; ^3^ Biomedical NMREindhoven University of TechnologyEindhoventhe Netherlands; ^4^ Laboratory of Biomechanical EngineeringUniversity of TwenteEnschedethe Netherlands; ^5^ Biomedical Engineering and PhysicsAcademic Medical CenterAmsterdamthe Netherlands; ^6^ Department of RadiologyUniversity Medical CenterUtrechtthe Netherlands

**Keywords:** DT‐MRI, fascicle length, fibertracking, MRI, segmentation, skeletal muscles

## Abstract

Musculoskeletal (dys‐)function relies for a large part on muscle architecture which can be obtained using Diffusion‐Tensor MRI (DT‐MRI) and fiber tractography. However, reconstructed tracts often continue along the tendon or aponeurosis when using conventional methods, thus overestimating fascicle lengths. In this study, we propose a new method for semiautomatic segmentation of tendinous tissue using tract density (TD). We investigated the feasibility and repeatability of this method to quantify the mean fascicle length per muscle. Additionally, we examined whether the method facilitates measuring changes in fascicle length of lower leg muscles with different foot positions. Five healthy subjects underwent two DT‐MRI scans of the right lower leg, with the foot in 15° dorsiflexion, neutral, and 30° plantarflexion positions. Repeatability of fascicle length measurements was assessed using Bland–Altman analysis. Changes in fascicle lengths between the foot positions were tested using a repeated multivariate analysis of variance (MANOVA). Bland–Altman analysis showed good agreement between repeated measurements. The coefficients of variation in neutral position were 8.3, 16.7, 11.2, and 10.4% for soleus (SOL), fibularis longus (FL), extensor digitorum longus (EDL), and tibialis anterior (TA), respectively. The plantarflexors (SOL and FL) showed significant increase in fascicle length from plantarflexion to dorsiflexion, whereas the dorsiflexors (EDL and TA) exhibited a significant decrease. The use of a tract density for semiautomatic segmentation of tendinous structures provides more accurate estimates of the mean fascicle length than traditional fiber tractography methods. The method shows moderate to good repeatability and allows for quantification of changes in fascicle lengths due to passive stretch.

## Introduction

The architectural parameters of muscle‐tendon units determine the function that these have within the musculoskeletal system. Among these parameters we find, most importantly: the optimal fiber length, the physiological cross‐sectional area (PCSA) and the pennation angle. For instance, fascicles within long muscle‐tendon units can sustain longer excursions during daily activities than short muscles having a large PCSA, which are instead optimized to produce high forces within a limited range of fascicle lengths (Li et al. 2014).

It is well‐known that these architectural properties may change due to (patho‐)physiological conditions such as aging, exercise, disease, or surgical intervention (Kumagai et al. 2000; Sinha et al. 2015) which has an influence on muscle function (Kumagai et al. 2000; Reeves et al. 2004). For example, it was shown that muscle fascicle length decreases due to inactivity and lengthens after exercise, but it has also been shown that fascicle length directly correlates with performance in athletes (Kumagai et al. 2000; Narici et al. 2003; Reeves et al. 2004). Therefore, obtaining architectural properties such as fascicle length in a reliable fashion is of vital importance to understand skeletal muscle function and alterations therein due to (patho‐)physiological conditions.

Quantitative values for architectural parameters, are often estimated from available data obtained on dissected cadaveric specimens or from bright mode ultrasound (Ward et al. 2009; Kwah et al. 2013; Lee et al. 2015). However, these methods have serious limitations. Cadaveric material does not provide patient‐specific information. Ultrasound is limited to superficial muscles and offers mostly 2D measurements in a narrow field‐of‐view (FOV) (Kwah et al. 2013). A few studies exploited conventional anatomical magnetic resonance imaging (MRI) to derive muscle architecture (Albracht et al. 2008; Böl et al. 2011). Anatomical T_1_‐ and T_2_‐weighted scans provide sufficient contrast for quantification of muscle volume (Albracht et al. 2008; Smeulders et al. 2010), but fascicle orientations and pennation angles cannot be inferred from these scans.

Muscle fascicle architecture characterization by Diffusion‐Tensor MRI (DT‐MRI) does not suffer from the above‐mentioned shortcomings, as it can be applied to quantify subject specific in vivo 3D muscle architecture in a large FOV (Sinha and Yao 2002; Kermarrec et al. 2010). DT‐MRI is a specialized MRI technique, capable of quantifying the self‐diffusion of water molecules in tissue. Water diffusion in skeletal muscles is highest along the axis of the fascicles and lowest perpendicular to the axis of the fascicles. Although a definitive model underlying this diffusion anisotropy in skeletal muscle is lacking, it is generally accepted that diffusion perpendicular to the muscle fascicle axis is lower because water diffusion in this direction is hindered by intra‐ and extracellular tissue constituents (Budzik et al. 2014; Oudeman et al. 2016). Using the diffusion tensor the muscle fascicle orientation can be quantified in each imaging voxel. Tractography combines this information in 3D to obtain whole‐muscle fascicle architecture (Oudeman et al. 2016). DT‐MRI and tractography have been applied to visualize muscle architecture in various regions in the human body, including leg, forearm, heart, spine, pelvis, and tongue (Gilbert and Napadow 2005; Heemskerk et al. 2009; Froeling et al. 2012; Zijta et al. 2012; Sieben et al. 2013).

It has been shown that tractography provides a useful visual representation of muscle architecture in which pennation angles could be measured accurately (Heemskerk et al. 2010; Schenk et al. 2013). However, the measurements of fascicle length proved more challenging due to the presence of artificially long fascicles as tractography continued beyond the muscle origin and insertion due to partial volume effects with tendons and fascia (Schenk et al. 2013; Sinha et al. 2015). Additionally, unrealistically short fascicles were observed near the borders of the segmented muscle volumes as a result of suboptimal segmentation and limited resolution (Schenk et al. 2013).

Thus, accurate and automatic determination of fascicle lengths and pennation angles not only depends on accurate segmentations of the individual muscles, but also on the accurate segmentation of tendons, fasciae, and (internal) aponeuroses (Heemskerk et al. 2010; Sinha et al. 2015). However, due to their limited resolution, segmentation cannot be performed reliably on DT‐MRI images (Budzik et al. 2014; Oudeman et al. 2016), therefore, segmentation has to be performed on high‐resolution coregistered anatomical scans. While this process is laborious and time consuming, it is also prone to errors due to difficulties in achieving a perfect registration between the anatomical and DT‐MRI scans.

The purpose of this work is to introduce a novel method for semiautomatic segmentation of tendinous structures directly from the DT‐MRI data, facilitating accurate and repeatable quantification of muscle fascicle lengths without the need for laborious segmentation and avoiding registration errors. We have explored this new method to quantify muscle fascicle lengths in several muscles of the human lower leg and we assessed changes in fascicle length due to passive ankle motion.

## Methods

### MRI

Five healthy male volunteers were scanned with a 3T Achieva MRI scanner (Philips, Best, the Netherlands) using a 6 channel torso coil. This study was approved by the local IRB and written informed consent was provided by all subjects prior to the study. A custom‐built device was used to immobilize the foot in 3 different positions: 15° dorsiflexion, neutral position, and 30° plantarflexion. MRI measurements of the lower leg for all three foot positions were performed in one examination. Each subject was measured twice on the same day in two separate MRI sessions, with at least 30 min in between. The MRI protocol consisted of an mDixon scan, for anatomical reference, and a DT‐MRI scan. The following scan parameters were used for 3‐point mDixon: sequence = FFE, FOV = 192 × 156 mm^2^, TR = 7.7 msec, TE1/ΔTE = 2.1/1.7 msec, matrix size = 192 × 192, number of slices = 100, voxel size = 1 × 1 × 2.5 mm^3^. The following scan parameters were used in the DT‐MRI scan: sequence = SE‐EPI, FOV = 192 × 156 mm^2^; TR = 11191 msec, TE = 51.63 msec, matrix size = 64 × 52, number of slices = 50, voxel size = 3 × 3 × 5 mm^3^, SENSE acceleration factor = 1.5, number of gradient directions = 12, diffusion *b*‐value = 400 sec/mm^2^; Slice‐selection gradient reversal (SSGR) was used for fat suppression, in combination with spectrally adiabatic inversion recovery (SPAIR) for aliphatic fat suppression and spectrally selective suppression of the olefinic fat peak (Hooijmans et al. 2015). The scan time for each foot position was 11 min, resulting in a total scan time of 33 min per session. Additionally, for each DT‐MRI scan a noise scan was also performed in order to calculate SNR maps.

### Data processing

#### Tensor calculation

DT‐MRI data were processed using DTITools for Mathematica 10.3 (Froeling et al. 2012). Data preprocessing comprised three steps: (1) Rician noise suppression (Froeling et al. 2009); (2) affine registration of the diffusion‐weighted images to the nonweighted image to correct for motion and eddy current deformations (Leemans and Jones 2009); and (3) b‐spline registration of the diffusion data to the mDixon water images to correct for susceptibility induced EPI deformations (Wu et al. 2008; Irfanoglu et al. 2011, 2012). In the last processing step, the resolution of the diffusion data was increased to a voxel size of 1.5 × 1.5 × 3.0 mm^3^. Directional diffusion data were fitted to a tensor model using a Weighed Least Linear Square (WLLS) algorithm, from which the principal direction of diffusion was determined. SNR (Signal to Noise Ratio) was defined as the ratio of the mean muscle signal in the DT‐MRI images acquired with *b* = 0 sec/mm^2^ and the standard deviation of noise, calculated from the noise scan.

#### Muscle segmentation

Muscle segmentation was done by manual delineation of the 11 muscles in the lower leg (i.e., Tibialis Anterior, Tibialis Posterior, Extensor Digitorum Longus, Flexor Digitorum Longus, Flexor Hallucis Longus, Extensor Hallucis Longus, Gastrocnemius Medialis, Gastrocnemius Lateralis, Soleus, Fibularis Brevis and Fibularis Longus). The delineation was performed in the out‐of‐phase mDixon images of the first measurement session with the foot in neutral position. These mDixon images were first down‐sampled to a resolution of 1 × 1 × 10 mm^3^, which resulted in only 25 slices for delineation. The segmentations were then transformed to all six datasets (3 positions, 2 measurements) by registering the down‐sampled out of phase mDixon images to full resolution out‐of‐phase mDixon images using rigid registration followed by nonlinear b‐spline registration with Elastix (Klein et al. 2010).

#### Tractography

Muscle tractography was performed using the vIST/e toolbox (http://bmia.bmt.tue.nl/software/viste). Tractography was performed with an allowed FA range of 0.1–0.7, a maximal allowed angle change per step‐size of 20° and a minimal fiber length of 0.2 cm. Seed points from which tracts continued bidirectionallly were evenly spaced throughout the whole leg volume (seed distance = 1 mm) and a deterministic algorithm was used for tractography. This initial whole volume tractography was subsequently used to determine tract density maps, as explained in the next section.

### Tract density and fascicle length

#### Tract density

**Figure 1:**
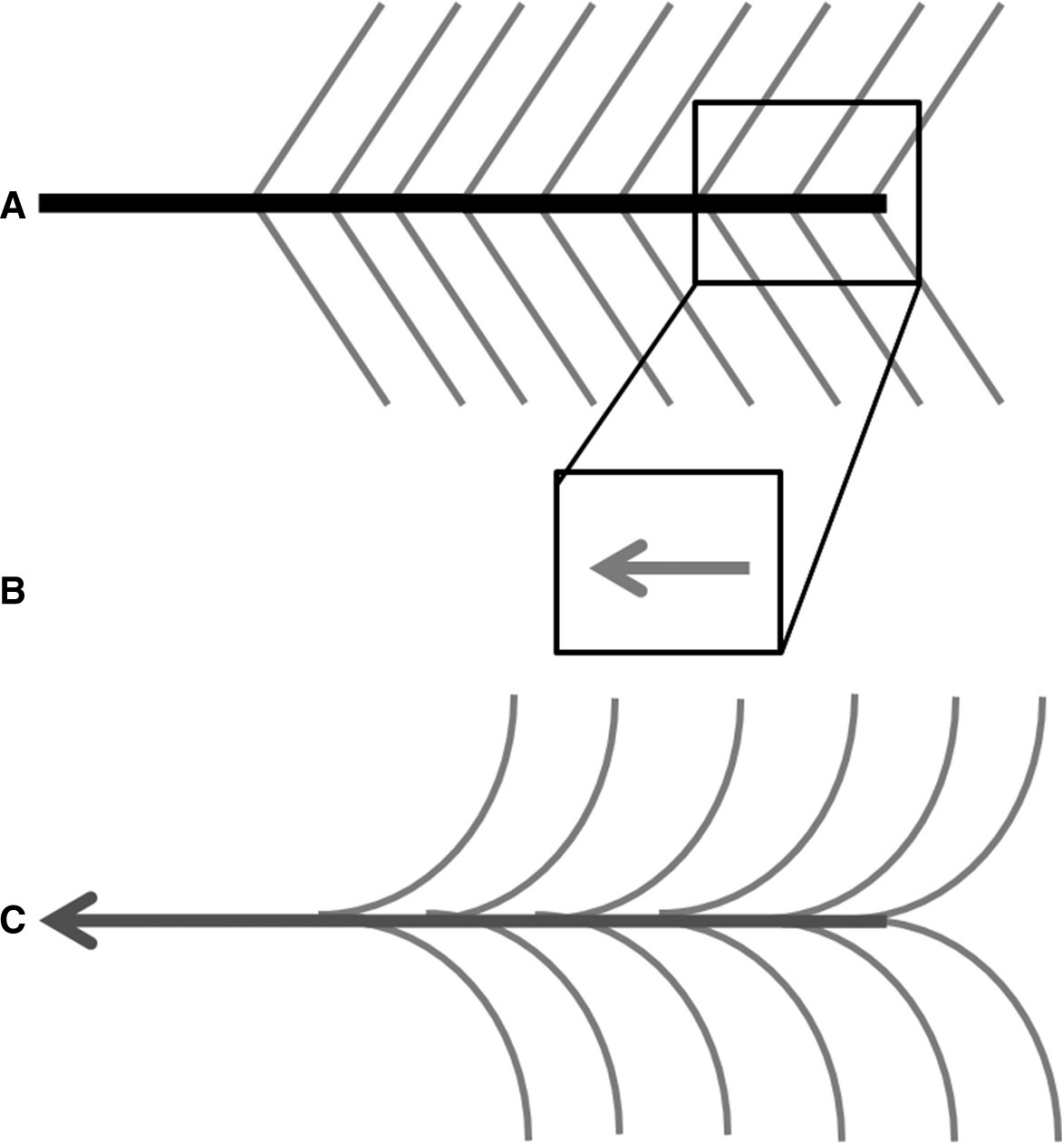
Schematic drawings of muscle and tendon in a pennate muscle with the artificial elongation. (A) The in vivo situation in which the muscle fascicles (gray) attach to the tendon (black). (B) When the voxel contains both tendon and muscle the average direction for tracking is along the tendon. (C) Therefore tractography will continue along the tendon, leading to artificially long fascicles.

The automatic segmentation of tendinous tissue is based on the notion that most muscle fibers have a well‐defined origin and insertion and have a relatively constant density in the muscle body. As a consequence, the tract density (TD) of reconstructed muscle fascicles – from DT‐MRI – should remain constant within the muscle volume (Fig. 1A). However, muscle fascicle tracts reconstructed from DT‐MRI data may artificially extend along tendons, aponeuroses, and fasciae, due to partial volume effects and diffusion anisotropy in these tendinous tissues (Fig. 1B). As a consequence, the fascicle TD in voxels containing tendinous tissue is higher than those in the muscle belly (Fig. 1C). Thus, voxels containing tendinous tissue can be segmented based on their higher TD value as compared to muscle tissue.

#### Tractography based on tract density

TD maps were made by volume seeding of tracts in the whole leg with a seeding distance of 1 × 1 × 1 mm^3^. TD was defined as the number of tracts crossing each voxel. The TD values were normalized to the mean TD of the entire volume, which contains mostly muscles, consequently TD ≈ 1 for muscle tissue. After the construction of the TD maps, a second tractography step is performed for each individual muscle, with step length = 0.2 max angle/step = 10°, and minimum fiber length = 20 mm, using the TD value as stopping criterion. If the TD value exceeds 1.5 tractography is halted, because this indicates the presence of a tendon, aponeurosis, fascia, or artifacts. Seed points for tractography based on TD values were equally spaced within a volume obtained by eroding the segmented volume for each muscle to about 90% of its original size.

#### Fascicle length measurements

The mean fascicle length for each muscle was derived from a fit of a skewed Gaussian distribution to the fascicle length distribution of all tracts of that muscle. Reconstructed fascicle tracts that terminated proximally or distally at the edges of the FOV were excluded from analysis, since these do not represent the full muscle fascicle length. As a comparison, our new method using TD maps was compared to two conventional tractography methods for determining fascicle lengths in the TA muscle. The first method used FA as a tracking stopping criterion with an allowed range of FA = 0.15–0.65 (Sinha et al. 2011, 2015; Okamoto et al. 2012). The second one involved an accurate manual segmentation of the TA muscle and halting tractography at segmented boundaries of the TA muscle (Heemskerk et al. 2005; Khalil et al. 2010; Schenk et al. 2013).

In‐depth analysis of fascicle lengths and changes therein upon passive plantar‐ and dorsiflexion was restricted to the soleus (SOL), fibularis longus (FL), extensor digitorum (EDL), and the tibialis anterior (TA). Results for all muscles are reported in the supplemental material.

### Statistical analysis

Repeatability of fascicle length measurements of the SOL, FL, EDL, and TA was investigated using Bland–Altman plots and reported as the coefficient of variation (CV). CV is defined as 100%*SD/Mean, where SD is the standard deviation of the paired difference and Mean is the mean value calculated for the two repeated datasets. The CV is reported per muscle and position as well as for the four muscles combined. The minimal detectable difference (MDD) is calculated for each of the four muscles. The MDD is equal to 1.96 times the SD of the paired differences and represents the smallest difference in fascicle length that, with a 95% confidence interval, can be attributed to a true change in fascicle length rather than to a measurement error.

Significance (*P *<* *0.05) of changes in parameter values between the three ankle positions and repeated measurements were tested with a multivariate analysis of variance (MANOVA) (SPSS v. 22; IBM, Armonk, NY) with a Bonferroni post hoc test. For each variable, the assumption of sphericity was tested with the Mauchly test. If the sphericity assumption was violated, one of three corrections was used based on the Mauchly test output: the Greenhouse‐Geisser, the Huynh‐Feldt, or the lower‐bound correction.

## Results

**Figure 2:**
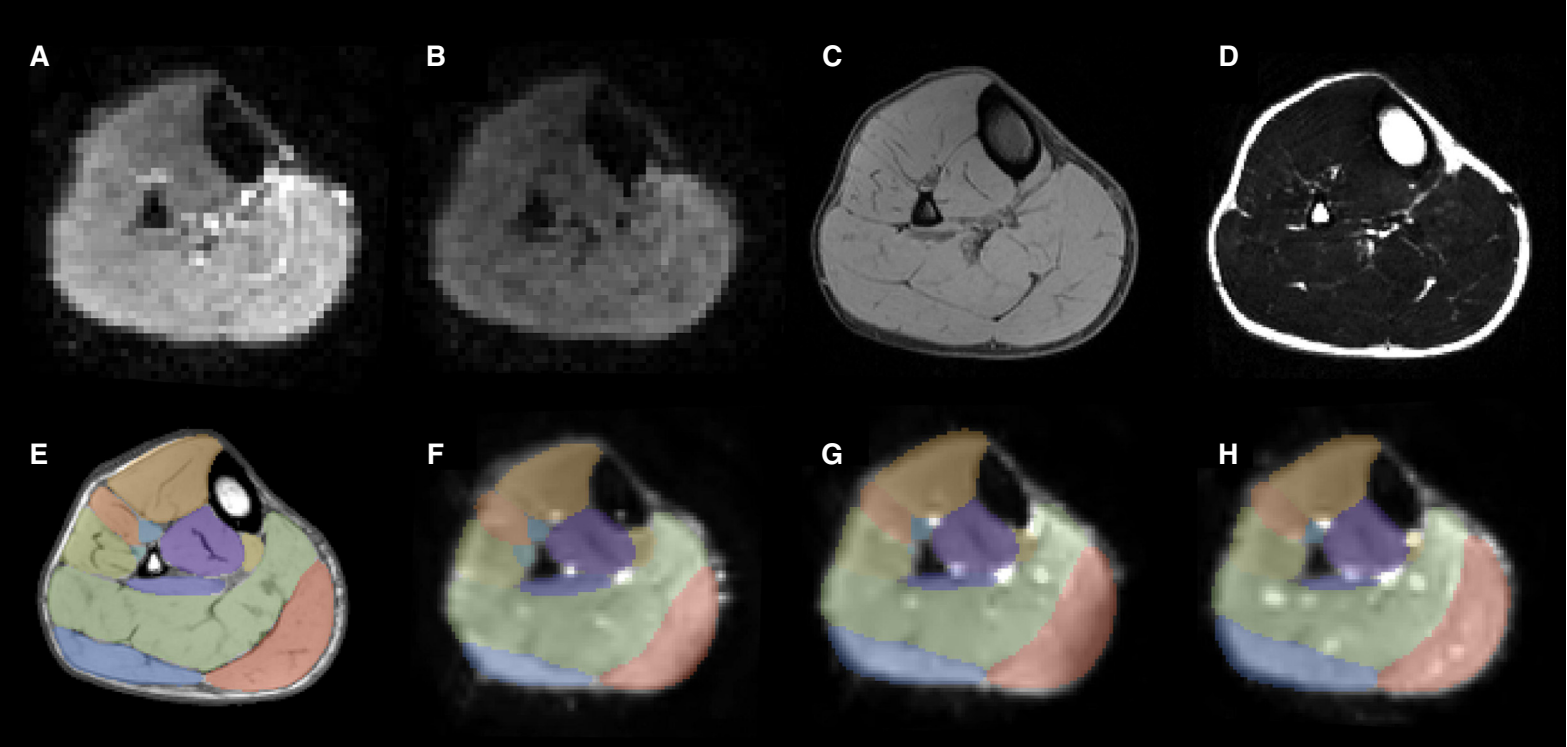
Transverse slice in a volunteer: raw MRI data and muscle segmentation. (A) DT‐MRI image obtained with b = 0 sec/mm^2^. (B) DT‐MRI image obtained with b = 400 sec/mm^2^. (C) Water image from the mDixon scan. (D) Fat image from the mDixon scan. (E) Muscle segmentation overlaid to the out‐of‐phase mDixon image. (F, G, H) Muscle segmentation obtained using the down‐sampled out‐of‐phase mDixon images overlaid to the up‐scaled diffusion data. (F) 15° dorsiflexion; (G) Neutral position; (H) 30° plantarflexion.

The five healthy males which were included in this study had a mean age of 27 years (range 24–29 years), a weight of 78 kg (range 62–89 kg), a length of 181 cm (range 171–189 cm), and a tibia length of 42 cm (range 37–47 cm). All DT‐MRI scans were completed successfully (see Fig. 2A–D) and were of sufficient quality to allow accurate tensor calculation. For all scans, the average signal‐to‐noise (SNR) of muscle in the nonweighted images was at least 30 (range 30–70). For all subjects the muscle segmentation using the down‐sampled out‐of‐phase mDixon images could be well aligned with the up‐scaled diffusion data (see Fig. 2E–H).

**Figure 3:**
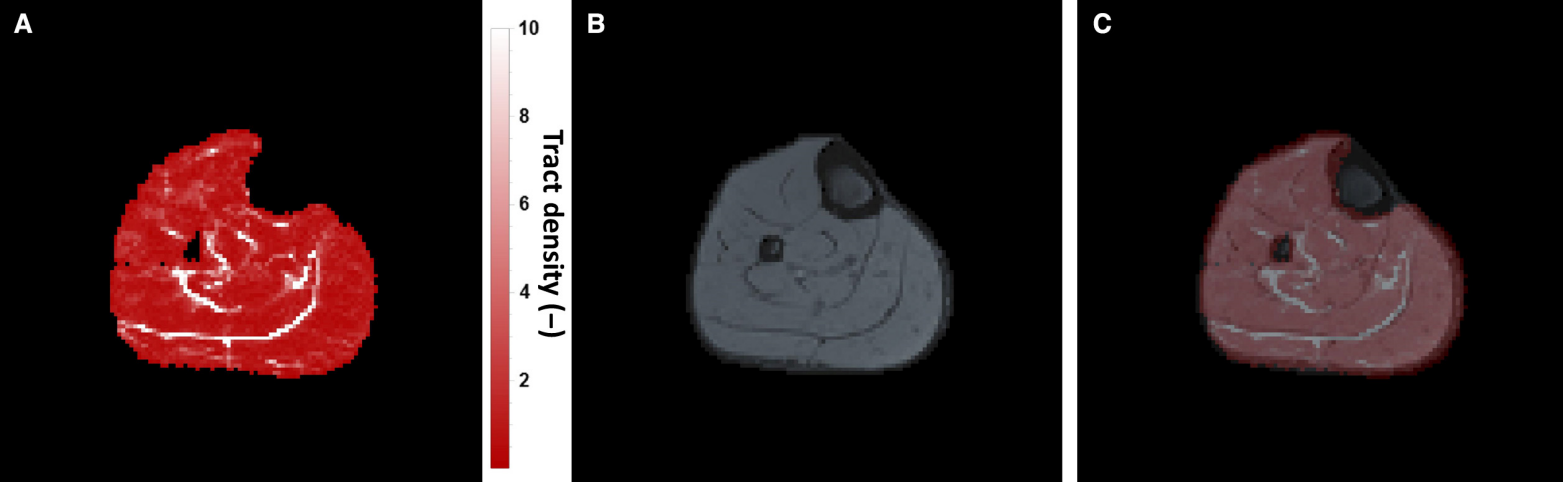
Tract density (TD) map and corresponding anatomical image. (A) Axial tract density map of the lower leg of one subject in neutral position. High values of normalized tract density are represented in white and indicate tendons, whereas low values in red can be assigned to muscle tissue. (B) Water image determined from the mDixon scan and (C) the previous two images overlaid. There is good anatomical agreement for the tendons between the two images.

Figure 3A is a representative TD map of the lower leg in axial orientation. The image shows that muscle tissue has constant TD values (red; TD ≈ 1 by normalization). Boundaries between muscles are characterized by pixels with considerably higher TD values (white; TD > 1.5). Figure 3B is the mDixon water image of the same slice, in which tendons and fasciae are hypo‐intense. The overlay of TD on the mDixon water image (Fig. 3C) shows that high TD pixels spatially correspond to mDixon water hypointense pixels. High TD pixels can thus generally be ascribed to tendons and fasciae, with the exception of artifacts or low SNR regions.

**Figure 4:**
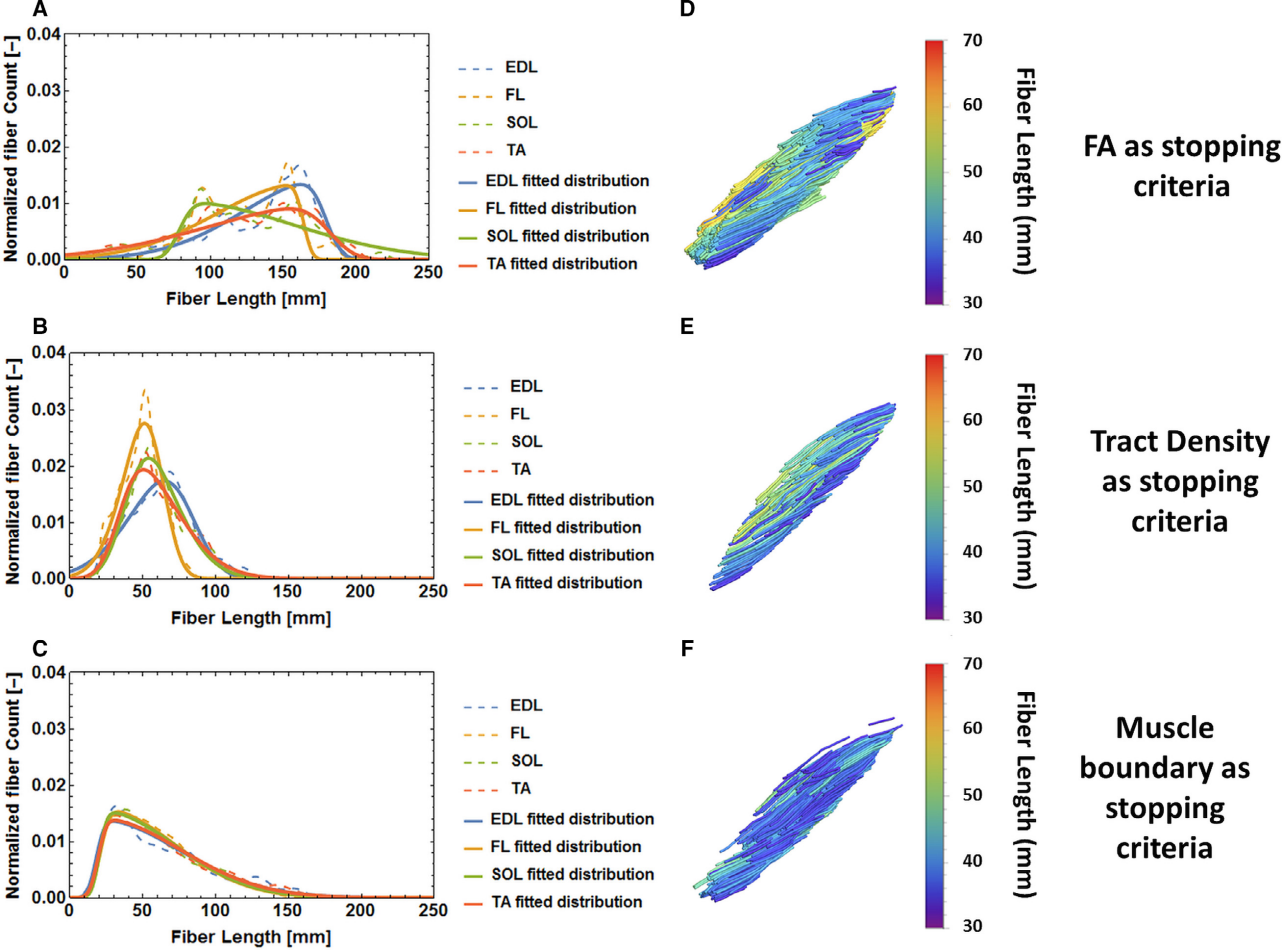
Comparison between tractography based on Tract Density, on FA and on muscle boundary. (A, C, E) Histograms of fascicle length in 4 muscles (Extensor digitorum longus (EDL), fibularis longus (FL), soleus (SOL) and tibialis anterior (TA)) in one volunteer with the foot in neutral position, using tracking stopping criteria based on FA, TD, and muscle segmentation volume, respectively. The dotted lines represent the histogram of fascicle count, and the solid line indicates the fitted distribution. Values of fascicle count are normalized so that the total area under the curve is equal to 1. (B, D, F) Tractography in the Tibialis Anterior colorcoded by fascicle length (in mm) in the same volunteer in neutral position.

Comparisons of tractography methods with different stopping criteria, including TD, are shown in Figure 4. When comparing the conventional tractography methods, which are based on FA or using the muscle boundary as stopping criteria, it can be seen that non‐Gaussian distributions of muscle fascicle lengths are obtained. When using FA as the sole stopping criteria, fascicles tend to extend into adjacent muscles and tendons (Fig. 4B). As a consequence, the mean value of fascicle length is shifted toward artificially larger values (Fig. 4A). When using muscle boundaries as a cutoff, the reconstructed fascicle lengths near the muscle boundary terminate prematurely (Fig. 4F), resulting in a skewed distribution toward short fascicle lengths (Fig. 4E). Using the TD‐based method, both long and short fascicles are avoided (Fig. 4D) and a normal distribution can be appreciated for all investigated muscles (Fig. 4C).

**Figure 5:**
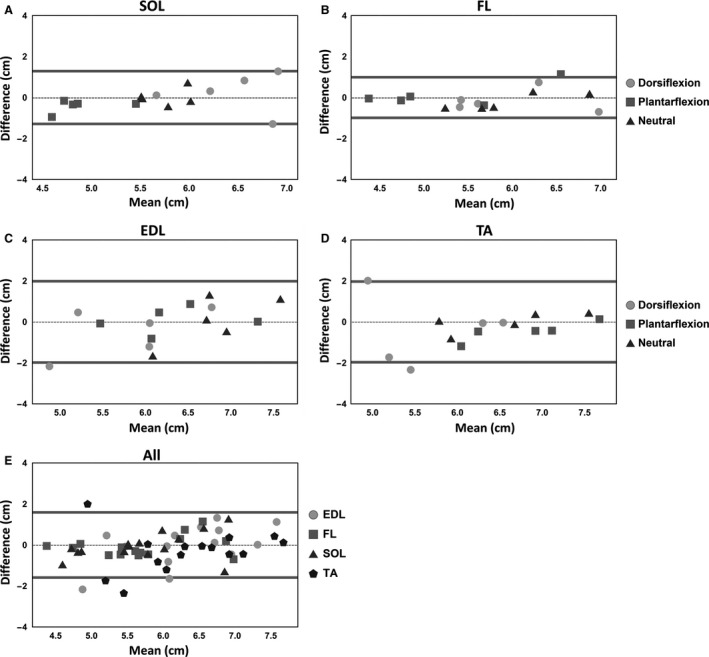
Bland–Altman of fascicle length measured in two different sessions. (A–D) Bland–Altman plots for the soleus (SOL), fibularis longus (FL), extensor digitorum longus (EDL), and tibialis anterior (TA). (E) Combined Bland–Altman plot for the four muscles. The dashed lines represent the mean difference, and the solid lines indicate 1.96 times the standard deviation.

Bland–Altman plots for fascicle length for SOL, FL, EDL, TA, and for all muscle together are shown in Figure 5. The graphs show that the differences between the two repeated measurements are distributed around a mean value of zero, indicating the absence of any systemic differences between sessions. Different limits of agreement were observed for different muscles, with the SOL showing overall the best reproducibility and TA showing the worst (see also Bland–Altman plots of all muscles in supplemental material). The CVs were 8.3, 16.7, 11.2, 10.43% in the neutral position, for the SOL, FL, EDL, and TA, respectively (see Table 1). The CVs and MDDs per muscle and position are listed in Table 1, showing a broad range for CVs (5.3–18.7%) with the repeatability being worst in dorsiflexion.

**Table 1:** Mean values of fascicle length and intersession repeatability

		SOL	FL	EDL	TA
Dorsiflexion	Mean ± SD (cm)	6.4 ± 0.7	5.9 ± 0.7	5.8 ± 0.9	5.7 ± 0.7
	CV (%)	10.6	10.5	16.3	18.7
	MDD (cm)	1.3	0.8	1.7	2.3
Neutral	Mean ± SD (cm)	5.7 ± 0.3	5.9 ± 0.6	6.8 ± 0.8	6.6 ± 0.7
	CV (%)	8.3	16.7	11.2	10.4
	MDD (cm)	0.4	0.8	0.9	0.6
Plantar flexion	Mean ± SD (cm)	4.9 ± 0.4	5.2 ± 0.9	6.3 ± 0.7	6.8 ± 0.6
	CV (%)	5.3	11.8	11.2	11.1
	MDD (cm)	0.6	0.6	1.7	0.7

Mean and standard deviation (SD) of the fascicle length, coefficient of variation (CV) and minimal detectable difference (MDD) per position indicated for four different muscles. SOL, soleus; FL, fibularis longus; EDL, extensor digitorum longus; TA, tibialis anterior.

**Figure 6:**
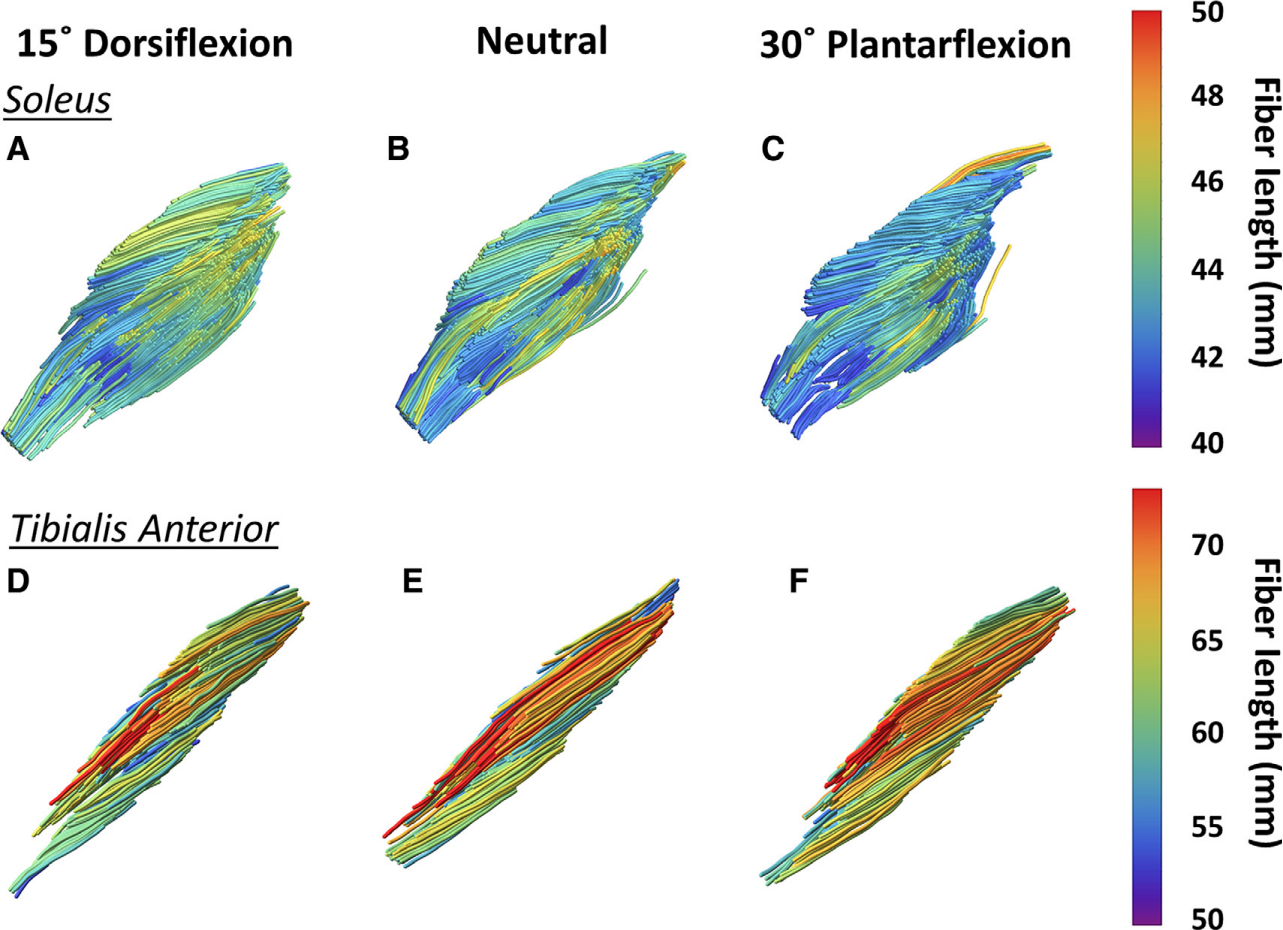
Fascicle tracts in soleus and Tibialis Anterior muscle color‐coded by fascicle length. Tractography in (A and D) dorsiflexion, (B and E) neutral, and (C and F) plantarflexion for the (A–C) soleus and (D–F) for the Tibialis Anterior of one subject. No to very little artificially long and short fascicles were reconstructed. Differences in fascicle lengths are visible from the different color of the fascicles in the three positions.

**Figure 7:**
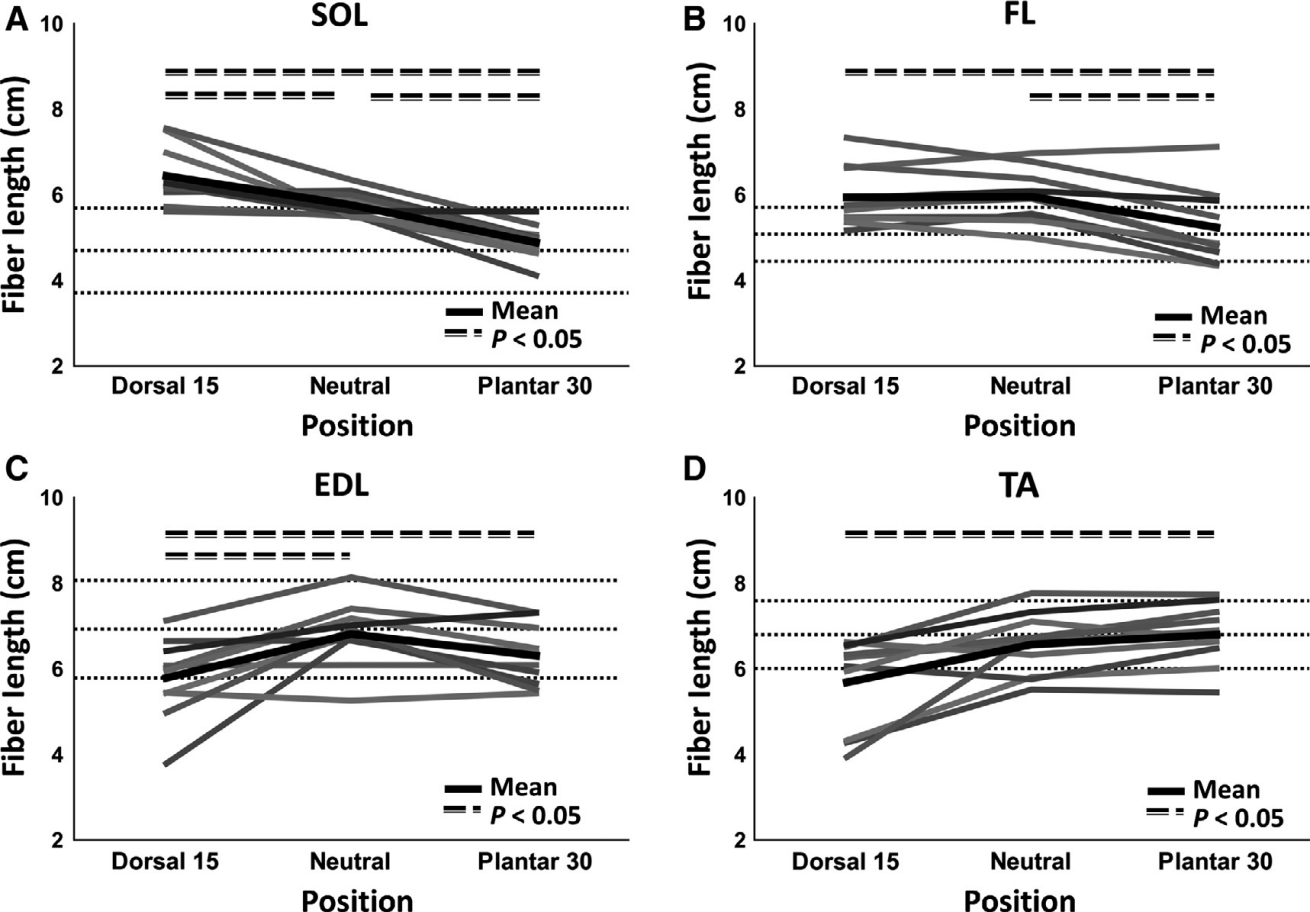
Mean muscle fascicle length in dorsiflexed, neutral, and plantarflexed foot position. Length (cm) plotted as a function of foot position for all separate scans for the soleus (SOL), fibularis longus (FL), extensor digitorum longus (EDL), and the tibialis anterior (TA). The mean value per muscle is indicated with the thick black line and the dotted lines indicate the mean and standard deviation found in literature (Ward et al. 2009). Furthermore, significance (*P *< 0.05) is shown by the thick straight dotted lines above the data.

Figure 6 shows the fascicles of the SOL and TA color‐coded for fascicle length in the three positions. In this figure the fascicle length changes can be appreciated. Furthermore, none to very little artificially elongated or shortened fascicles are seen. The values of average fascicle lengths for SOL, FL, EDL, and TA and different positions are presented in Table 1. Figure 7 shows the fascicle length as a function of ankle position for SOL, FL, EDL, and TA per subject; the black line indicates the mean calculated over the 10 measurement. For the graphs of all muscles see supplemental material. Significant (*P *<* *0.05) change in the fascicle length was observed from dorsiflexion to neutral for SOL and EDL, from neutral to dorsiflexion for SOL, FL, and EDL. From dorsiflexion to plantarflexion the change was significant for SOL, FL, EDL, and TA (Fig. 7).

## Discussion

In this work, we introduced a novel method for automatic segmentation of tendinous structures directly from DT‐MRI data, facilitating accurate and repeatable quantification of muscle fascicle lengths. The resulting fascicle lengths, obtained by excluding tendinous structures from tractography, are in agreement with previously reported muscle fascicle lengths in cadaveric specimens (Ward et al. 2009). Furthermore, fascicle length measurements showed good to moderate repeatability (CV of 5.3–18.7%) and quantification of changes in fascicle length due to changes in ankle position proved possible.

The proposed method effectively avoids the reconstruction of artificially long and short fascicles, and the overall normalized counts of fascicle length within a muscle approached a Gaussian distribution, which is agreement with findings in cadaveric material (Schenk et al. 2013). The problem of artifically long fascicles along the aponeurosis and tendons, which bias the mean fascicle length toward higher values, was already addressed in a previous study by manual editing of fascicles (Sinha et al. 2015). While manually editing results in correct fascicle lengths, it is a very time consuming process. Furthermore, it requires accurate tendon segmentation from high‐resolution anatomical scans and/or careful visual inspection of all reconstructed fascicles. The strength of our method is that it facilitates automatic and user independent segmentation of tendons and estimations of muscle fascicle lengths.

The presented fascicle lengths are in agreement with literature (Klein Horsman et al. 2007; Ward et al. 2009; Kwah et al. 2013). Ward et al. (2009) reported fascicle length values for SOL, FL, EDL, and TA of cadaveric specimens in neutral position of 4.7 ± 1 cm, 5.1 ± 0.6 cm and 7.0 ± 1.1 cm and 6.8 ± 0.8 cm, respectively. We found average values of 5.7 ± 0.3 cm, 5.9 ± 0.6 cm, 6.8 ± 0.8 cm, and 6.6 ± 0.7 cm, respectively, in neutral position. Our results are also in agreement with ultrasound values of TA fascicle length in neutral position, which range from 5.5 to 8 cm (Chleboun et al. 2007; Klimstra et al. 2007; de Boer et al. 2008). However, most ultrasound studies have studied active contractions and small parts of the muscle only.

We found a wide range of reproducibility indices between muscles (see supplemental material) as well as between foot positions. In particular, an overall increased CV is observed for the dorsiflexed position, most likely caused by the fact that the position was not well tolerated by a few subjects resulting in some active involuntary muscle contraction. However, the repeatability of fascicle length measurements in our study is better compared to previous DT‐MRI studies (Heemskerk et al. 2010; Bolsterlee et al. 2015) in which only the neutral position for a single muscle was measured.

The changes in fascicle length with respect to neutral foot position obtained in this study are in agreement with the known antagonistic function of muscles. In fact, the TA and the EDL, that play a role in dorsiflexing the foot, present a reduced fascicle length in the dorsiflexed foot position, when compared to the plantar flexed position, with the biggest change between dorsiflexion and the neutral position, being significant for the EDL. On the other hand, the SOL and the FL muscles, which are plantarflexor muscles, present the opposite behavior, with significant shorter fascicles in the plantarflexed position compared to the dorsiflexed position, with the biggest change between the plantarflexed and neutral position and which was significant for both SOL and FL.

With the new method we measure realistic fascicle lengths for all muscles (see supplemental material). For some muscles, specifically for GCM and GCL, fascicle lengths changes from dorsi‐ to plantarflexion were small and not significant. We believe this is because for these muscles the change in length in the total muscle‐tendon complex is partially attributed to stretch of the tendon during passive motion. As shown by Herbert et al. (2002) the contribution of tendon stretch in the total variation in length of the muscle‐tendon complex is much larger for the GCM and GCL than for the TA. In individual cases, shortening of a muscle was observed where a lengthening was expected. We believe that this is in part due to tendon compliance, but mostly due to active contraction of these muscles in dorsiflexed position which was perceived as uncomfortable by some of the volunteers.

Nevertheless, our study shows that the new method can be used to obtain reliable patient‐specific fascicle lengths, which ultimately might be used as geometrical input for biomechanical models (Napadow et al. 2002; Mijailovich et al. 2010; Lee et al. 2015; Siebert et al. 2015). More important, our study shows fascicle length measurements can be applied to monitor disease, therapy, or training. As an example, previous studies reported that 90 days of bed rest caused a 10% decrease in fascicle length of the GCM and GCL (Reeves et al. 2002), opposing to an 11% increase in the fascicle length of the Vastus Lateralis muscles (VL) of the upper leg in elderly people after training (Reeves et al. 2004). Moreover, in competitive sprinting, fascicle length was found to be the main determinant of the performance (Kumagai et al. 2000). The reported changes in fascicle length observed in those studies were all in the range of the MDDs found in all of the muscles in our study, and therefore could also be determined using our new method.

Our method also opens the possibility to do longitudinal studies of the response in fascicle lengths to therapies or diseases. Furthermore, the automated tendon segmentation fascicle tractography could be extended with quantifications of pennation angles and muscle volumes. This should be possible in an automated fashion as we identified the voxels belonging to tendinous structures as well as the voxels which consist purely of muscle tissue.

### Limitations

This study has a few limitations. First of all, the number of subjects was limited to 5. However, for proof‐of‐concept and assessing repeatability of fascicle length quantification in various muscles in the lower leg, this proved sufficient. A second limitation was that the 15° dorsiflexion position was not always well tolerated and may have caused compensatory muscle contractions in some of the subjects. In general, complete passive stretch might be hard to achieve. Although our fascicle length estimates were in good agreement with cadaveric and ultrasound studies, we did not validate in vivo fascicle length measurement directly on the same muscles. This could be done in animal studies, where after in vivo measurements the muscle could be dissected for ex vivo validation.

Another potential limitation is the positioning of the subject with the calf resting on the coil, rather than being suspended. While this set‐up is favorable for maximizing SNR in the posterior compartment, it also leads to deformation and compression of the gastrocnemius muscles. If interested in these muscles, a set‐up with the calf suspended would be preferable (Lorbergs et al. 2015; Elzibak and Noseworthy 2013).

## Conclusions

In conclusion, we presented a novel in vivo approach for skeletal muscle fascicle length measurement using DT‐MRI. This method showed good to moderate repeatability and enabled quantification of changes in muscle fascicle lengths with passive stretch of lower leg muscles. Ultimately, validation of fascicle length estimations could be obtained by comparing post mortem DTI with anatomical dissection.

## Supporting information




**Figure S1.** Bland–Altman of fascicle length measured in two different sessions for all muscles of the calf.
**Figure S2.** Mean muscle fascicle length in dorsiflexed, neutral, and plantarflexed foot position for all muscles of the calf.
**Table S1.** Mean fascicle length and standard deviation (SD), coefficient of variation (CV) and minimal detectable difference (MDD) as a function of foot position for the tibialis posterior (TP), fibularis brevis (FB), extensor hallucis longus (EHL), lateral gastrocnemicus (GCL), flexor hallucis longus (FHL), medial gastrocnemicus (GCM) and flexor digitorum longus (FDL).
