# Supplementary material for: A novel diffusion‐tensor MRI approach for skeletal muscle fascicle length measurements
Source: Physiol Rep. 2016 Dec 21;4(24):e13012. doi: 10.14814/phy2.13012 (PMC5210383; doi:10.14814/phy2.13012)
Supplement: Supplementary file 1 — Figure S1. Bland–Altman of fascicle length measured in two different sessions for all muscles of the calf. Figure S2. Mean muscle fascicle length in dorsiflexed, neutral, and plantarflexed foot position for all muscles of the calf. Table S1. Mean fascicle length and standard deviation (SD), coefficient of variation (CV) and minimal detectable difference (MDD) as a function of foot position for the tibialis posterior (TP), fibularis brevis (FB), extensor hallucis longus (EHL), lateral gastrocnemicus (GCL), flexor hallucis longus (FHL), medial gastrocnemicus (GCM) and flexor digitorum longus (FDL). [file PHY2-4-e13012-s001.doc]

**A novel diffusion-tensor MRI approach for skeletal muscle fascicle length measurements. Supplemental Material**

Jos Oudeman,^1a,b)^, Valentina Mazzoli^1,2,3,b)^, Marco A. Marra^2^, Klaas Nicolay^3^, Mario Maas^1^, Nico Verdonschot^2,4^, Andre M. Sprengers^2,4^, Aart J. Nederveen^1^, Gustav J. Strijkers^5^, Martijn Froeling^6^

^1^ Department of Radiology, Academic Medical Center, Amsterdam, 1100DD, The Netherlands

^2^ Orthopedic Research Lab, Radboud UMC, Nijmegen, 6525 GA, The Netherlands

^3^ Biomedical NMR, Eindhoven University of Technology, Eindhoven, 5600 MB The Netherlands

^4^ University of Twente, Laboratory of Biomechanical Engineering, Enschede 7500 AE, The Netherlands

^5^ Biomedical Engineering and Physics, Academic Medical Center, Amsterdam, 1100DD, The Netherlands

^6^ Department of Radiology, University Medical Center, Utrecht, 3584 CX, The Netherlands

^a)^Corresponding author

E-mail: J.Oudeman@amc.uva.nl

^b)^These authors contributed equally


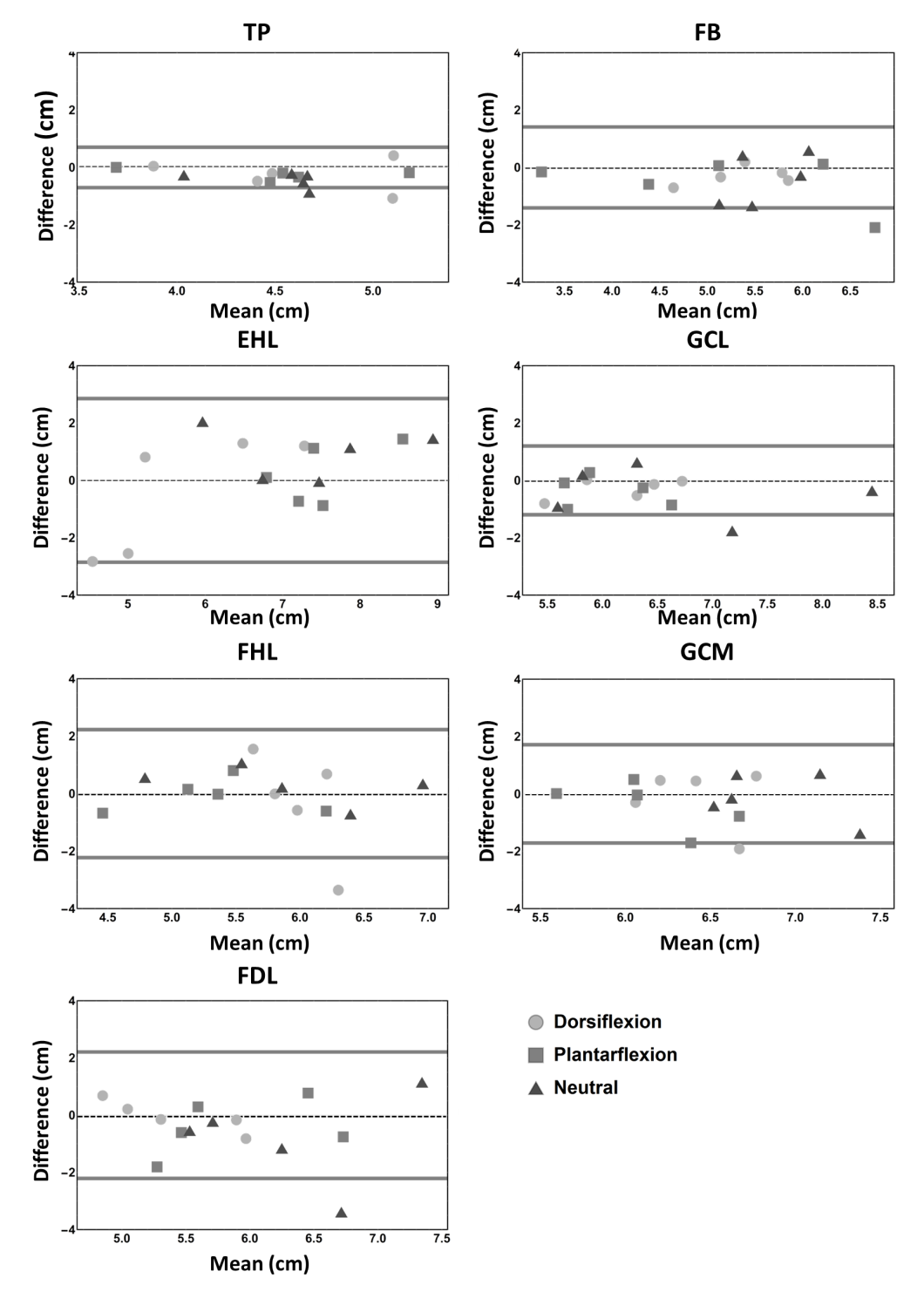


**Bland-Altman of fascicle length measured in two different sessions for all muscles of the calf.** Bland-Altman plots for the Tibialis Posterior (TP), Fibularis Brevis (FB), Extensor Hallucis Longus (EHL), Lateral Gastrocnemicus (GCL), Flexor Hallucis Longus (FHL), Medial Gastrocnemicus (GCM), Flexor Digitorum Longus (FDL). The dashed lines represent the mean difference, and the solid lines indicate 1.96 times the standard deviation.


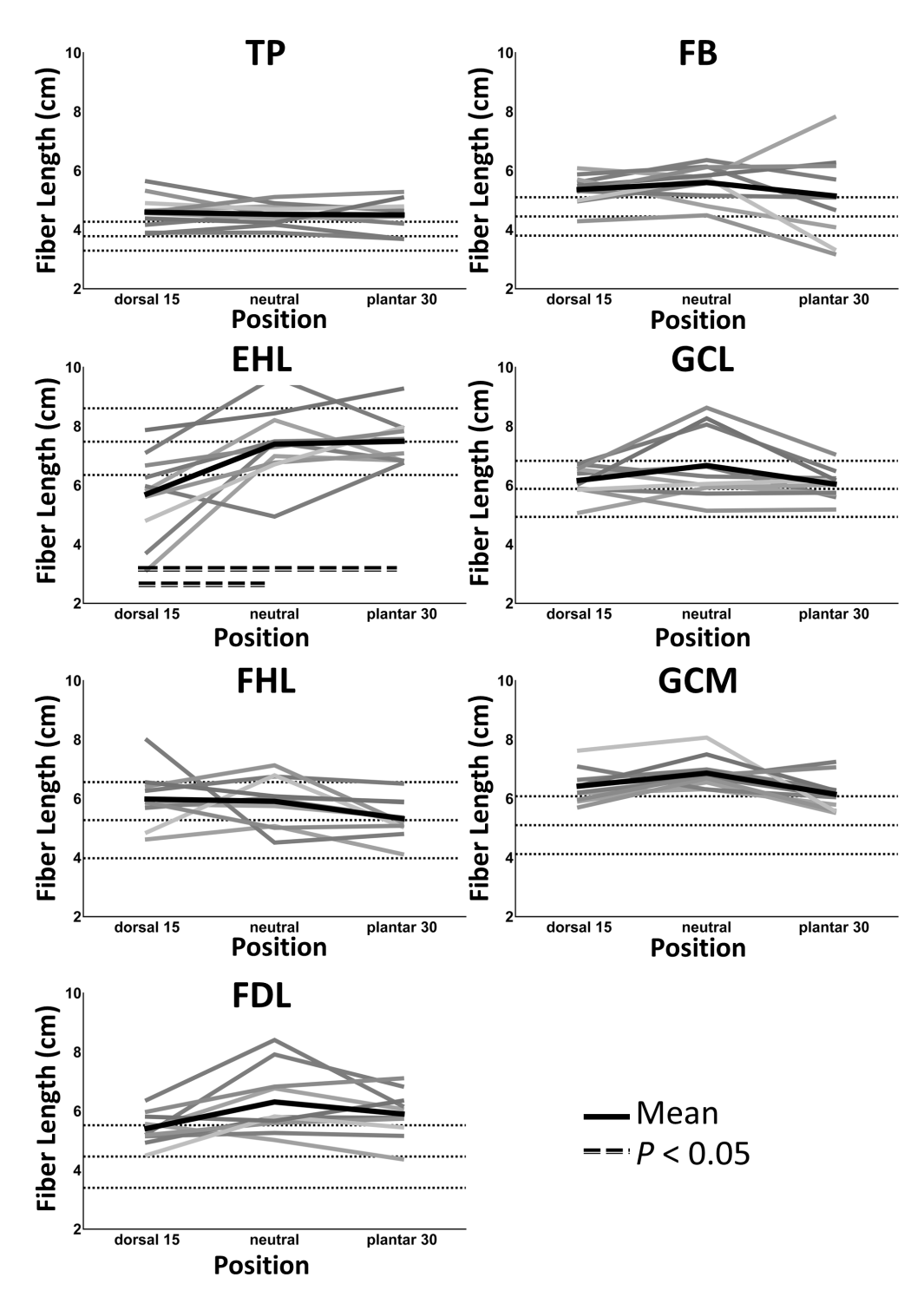


Mean muscle fascicle length in dorsiflexed, neutral and plantarflexed foot position for all muscles of the calf. Length (cm) plotted as a function of foot position for all separate scans for the Tibialis Posterior (TP), Fibularis Brevis (FB), Extensor Hallucis Longus (EHL), Lateral Gastrocnemicus (GCL), Flexor Hallucis Longus (FHL), Medial Gastrocnemicus (GCM), Flexor Digitorum Longus (FDL). The mean value per muscle is indicated with the thick black line and the dotted lines indicate the mean and standard deviation found in literature (49). Furthermore, significance (P < 0.05) is shown by the thick straight dotted lines above the data.

|  |  | ***Dorsiflexion*** | ***Neutral*** | ***Plantarflexion*** |
| --- | --- | --- | --- | --- |
| **TP** | Mean±SD (cm) | 4.6±0.6 | 4.5±0.4 | 4.5±0.5 |
|  | CV (%) | 12.5 | 11.8 | 8 |
|  | MDD (cm) | 0.8 | 0.3 | 0.4 |
| **FB** | Mean±SD (cm) | 5.4±0.5 | 5.6±0.6 | 5.2±1.4 |
|  | CV (%) | 9.7 | 27.6 | 10.8 |
|  | MDD (cm) | 0.5 | 1.3 | 1.3 |
| **EHL** | Mean±SD (cm) | 5.7±1.5 | 7.4±1.2 | 7.5±0.8 |
|  | CV (%) | 25.8 | 10.6 | 16.9 |
|  | MDD (cm) | 2.9 | 1.5 | 1.3 |
| **GCL** | Mean±SD (cm) | 6.2±0.5 | 6.7±1.2 | 6.0±0.5 |
|  | CV (%) | 8.5 | 8.7 | 18.1 |
|  | MDD (cm) | 0.5 | 0.7 | 1.3 |
| **FHL** | Mean±SD (cm) | 6.0±0.5 | 5.9±0.9 | 5.3±0.7 |
|  | CV (%) | 15.6 | 12.4 | 14.4 |
|  | MDD (cm) | 2.6 | 0.8 | 0.9 |
| **GCM** | Mean±SD (cm) | 6.4±0.6 | 6.9±0.5 | 6.2±0.6 |
|  | CV (%) | 9.0 | 9.7 | 7.9 |
|  | MDD (cm) | 1.5 | 1.2 | 1.2 |
| **FDL** | Mean±SD (cm) | 5.4±0.5 | 6.3±1.1 | 5.9±0.8 |
|  | CV (%) | 10.0 | 13.6 | 18.0 |
|  | MDD (cm) | 0.7 | 1.4 | 2.3 |

Supplemental table 1:
Mean fascicle length and standard deviation (SD), coefficient of variation (CV) and minimal detectable difference (MDD) as a function of foot position for the Tibialis Posterior (TP), Fibularis Brevis (FB), Extensor Hallucis Longus (EHL), Lateral Gastrocnemicus (GCL), Flexor Hallucis Longus (FHL), Medial Gastrocnemicus (GCM) and Flexor Digitorum Longus (FDL).
